# Potential PET tracers for imaging of tumor-associated macrophages

**DOI:** 10.1186/s41181-022-00163-2

**Published:** 2022-05-08

**Authors:** Bruna Fernandes, Paula Kopschina Feltes, Carolina Luft, Luiza Reali Nazario, Cristina Maria Moriguchi Jeckel, Ines F. Antunes, Philip H. Elsinga, Erik F. J. de Vries

**Affiliations:** 1 Department of Nuclear Medicine and Molecular Imaging, University Medical Center Groningen, University of Groningen, P.O. Box 30 001, 9700 RB Groningen, The Netherlands; 2 Graduate Program in Biomedical Gerontology, School of Medicine, Pontifical Catholic University of Rio Grande do Sul (PUCRS), Porto Alegre, Brazil; 3 Laboratory of Cellular Biophysics and Inflammation, Pontifical Catholic University of Rio Grande do Sul (PUCRS), Porto Alegre, Brazil; 4 Laboratory of Pediatric Physical Activity, Infant Center, Pontifical Catholic University of Rio Grande do Sul (PUCRS), Porto Alegre, Brazil

**Keywords:** Tumor-associated macrophages, Tumor microenvironment, M2-like, M1-like, PET imaging

## Abstract

The increasing incidence of cancer over the years is one of the most challenging problems in healthcare. As cancer progresses, the recruitment of several immune cells is triggered. Infiltration of tumor-associated macrophages (TAMs) is correlated with poor patient prognosis. Since TAMs constitute a big portion of the tumor mass, targeting these cells seems to be an attractive approach for cancer immunotherapy. Additionally, TAM assessment using non-invasive imaging techniques, such as positron emission tomography (PET), might provide a better understanding of the role of TAMs in cancer, and a means for tumor profile characterization, patient selection for individualized immunotherapy and treatment monitoring. Imaging of TAMs using PET tracers is still in its infancy. TAMs have several characteristics that could be exploited as potential targets for imaging. Various PET tracers for these TAM biomarkers have been developed, although often in the context of (neuro)inflammatory diseases rather than cancer. Since macrophages in inflammatory diseases express similar biomarkers as TAMs, these PET tracers could potentially also be applied for the assessment of TAMs in the tumor microenvironment. Therefore, the present review provides an overview of the TAM biomarkers, for which potential PET tracers are available and discusses the status of these tracers.

## Background

In the last decades, an exponential growth in new cases of cancer has been reported worldwide. Cancer is a leading cause of death and limits the increase in life expectancy in all countries of the world (Pilleron et al. 2019). In 2020, 19.3 million new cases were reported. The elderly population (65+ years old) accounted for 51.6% of new cases, whereas adults under 65 years old were responsible for 48.4%. Predictions expect a global cancer burden of approximately 28 million cases for the year of 2040, representing a 47% increase compared to 2020. The most common cancer types reported in 2020 were breast cancer, lung cancer, colorectum cancer, prostate cancer, stomach cancer, and liver cancer (Sung et al. 2021). Interestingly, all these cancers may present infiltration of macrophages (Skytthe et al. 2020; Quail and Joyce 2017).

Macrophages play a crucial role in the innate and adaptive immune system (Zhou et al. 2020). They express a broad phenotypic heterogeneity and functional diversity. The activation of macrophages is a stress-dependent process. Macrophages of the M0 phenotype (non-polarized) turn into the M1 phenotype (classically activated), when exposed to e.g. interferon-gamma (IFN-Ɣ), tumor necrosis factor (TNF) or lipopolysaccharide (LPS) (Ivashkiv 2018; Russell et al. 2019). Pro-inflammatory mediators such as reactive oxygen species (ROS), interleukin-12 (IL-12), IL-1β, TNF-α, IL-6, inducible nitric oxide synthase (iNOS), cyclooxygenase-2 (COX-2), among others, are released by these activated macrophages. In contrast, alternative activation by exposure to interleukin-4 (IL-4) and/or interleukin-13 (IL-13) results in the M2 phenotype, which is characterized by an increase in the release of anti-inflammatory mediators, such as interleukin-10 (IL-10) (Russell et al. 2019; Jung et al. 2019). Some authors have described that the phenotype shift goes beyond the plasticity dichotomy, where macrophages may even present two phenotypes at the same time depending on the moment of the injury, becoming the newly hypothesized M3 phenotype (Chistiakov et al. 2018; Malyshev and Malyshev 2015).

Besides the central role in tissue repair and systemic inflammation and immunity, macrophages play a crucial role in the tumor microenvironment (TME). Infiltrated macrophages in the TME, the so-called tumor-associated macrophages (TAMs), display broad phenotypic plasticity and can not only promote tumor regression (M1-like TAM), but also tumor progression (M2-like TAM). Since TAMs can represent approximately 30–50% of the tumor mass and trigger several factors that promote tumor growth, invasion, metastasis, and drug resistance (Quail and Joyce 2017; Malyshev and Malyshev 2015; Cendrowicz et al. 2021; Poh and Ernst 2018), they have been considered a potential target for early cancer detection and treatment (Quail and Joyce 2017). TAM-targeted therapies have gained considerable attention in the last years, resulting in the emergence of new drugs that can modulate and recalibrate the immune system within the TME. Therapeutic approaches such as depletion (e.g. with CSF-1R inhibitors), repolarization (e.g. with arginase inhibitors), inhibition of TAMs recruitment (e.g. inhibition of the CCR2 axis), and immune-checkpoint blockades (e.g. with PD-1 inhibitors) could improve treatment outcome (Li et al. 2021). Additionally, assessment of the infiltration levels and phenotype of TAMs might be helpful for the selection of the most suitable immune therapy (single or combined) for the patient and the evaluation of treatment response.

The present article aims to summarize the main TAM biomarkers that could be used as target for non-invasive prognosis and therapy response monitoring by means of Positron Emission Tomography (PET). PET is a state-of-the-art imaging technique that can provide in vivo insights in physiological and molecular processes, provided that a suitable imaging probe is available for the target of interest. The overview is limited to targets on TAM, for which PET tracers are already available, or in development.

## Main text

### Macrophages and the tumor microenvironment (TME)

Primary tumors and metastases are complex systems composed of neoplastic cells, extracellular matrix, and non-neoplastic cells, which include resident mesenchymal support cells, endothelial cells, and infiltrated immune cells (Gonzalez et al. 2018). The main immune cells involved in the initial inflammatory response are the neutrophils and macrophages, as well as dendritic, NK, and lymphoid cells (Pilleron et al. 2019; Cendrowicz et al. 2021; Mukherjee et al. 2019). When immune cell recruitment continues, an exacerbated activation occurs, leading to an unbalanced microenvironment, which leads to chronic inflammation and tumor growth (Chistiakov et al. 2018). During tumor development, angiogenesis and increased permeability of the vessels facilitate the supply of nutrients and oxygen to the TME (Chistiakov et al. 2018; Ngambenjawong et al. 2017; Murphy and Weaver 2016). TME is a dynamic and intricate system infiltrated not only by innate, but also by adaptative immune cells (Chistiakov et al. 2018; Murphy and Weaver 2016). T and B lymphocytes, NK cells, dendritic cells, macrophages, neutrophils, and myeloid-derived suppressor cells constitute the main immune cells in the TME that, in combination with other cells in the tissue, such as fibroblast, adipocytes, and vascular endothelial cells, trigger tumor progression (Galli et al. 2020).

TAMs can present up to 50% of tumor mass in solid tumors (Poh and Ernst 2018; Vinogradov et al. 2014). These cells play a key role in tumor progression, angiogenesis, and are associated with immunosuppression and the activation of a chronic inflammatory response (Poh and Ernst 2018). Thus, high levels of TAMs in the TME are usually associated with a poor prognosis of the disease (Poh and Ernst 2018; Chen et al. 2019).

#### TAM polarization

**Fig. 1:**
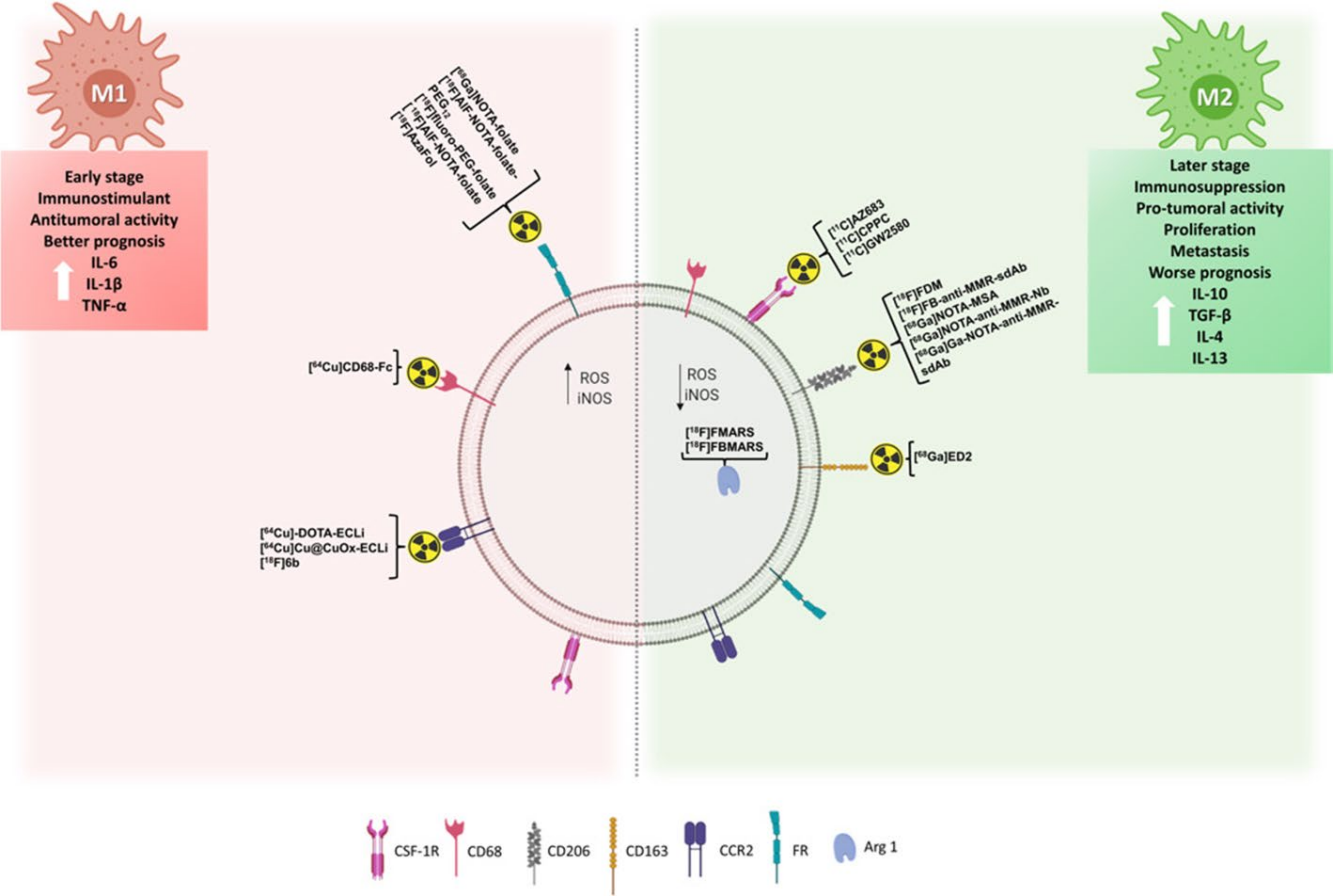
The main biomarkers expressed by TAM phenotypes, for which PET tracers are available. For each biomarker, the available PET tracers are indicated. CSF-1R: colony stimulating factor 1 receptor; CD: cluster of differentiation; CCR2: C–C chemokine receptor type 2; FR: folate receptor; Arg1: arginase 1; IL: interleukin; TNF-α: tumoral necrose factor α; TGF-β: transforming growth factor β

The polarization of the macrophages into the M1 and M2 phenotypes is generally associated with the stage of cancer (Fig. 1). The pro-inflammatory phenotype (M1) is often found in early stages, regressing or dead tissue-containing tumors (Boutilier and Elsawa 2021). These macrophages are responsible for the release of pro-inflammatory cytokines, such as INF-γ and TNF-α, and the activation of T cells as a tumoricidal mechanism (Boutilier and Elsawa 2021; Chen et al. 2018). M1 polarization through the macrophage migration inhibitory factor can also lead to tumor death by regulating both cell proliferation and invasiveness, activating the release of TNF-α and IL-1β (Poh and Ernst 2018; Chen et al. 2018). In contrast, tumors in more advanced stages mainly contain macrophages of the M2 phenotype, which induce a pro-tumoral state (tumor progression) (Boutilier and Elsawa 2021). Although the polarization mechanism of TAMs is not yet fully understood, studies show that a hypoxic environment and expression of hypoxia-inducible factors 1-α and 2-α are associated with increased expression of genes related to M2 polarization (Murphy and Weaver 2016). In addition, the secretion of anti-inflammatory cytokines by tumor cells is also an important factor in the polarization of M2 TAMs. The anti-inflammatory status characterized by M2 macrophages is related to immunosuppression (T-reg cells attraction), increased expression of programmed death-ligand 1 (PD-L1) on the surface of macrophages, the release of transforming growth factor-β (TGF-β), and secretion of cytokines such as IL-4 and IL-13 (Wang and DuBois 2015). Therefore, increased levels of M2-phenotype have been associated with pro-tumorigenic inflammation, leading to poor outcomes (Ngambenjawong et al. 2017).

### PET imaging and TAMs

PET imaging could be a useful tool for characterization of TAM polarization, depletion, and recruitment (inhibition). Noteworthy, most studies using PET tracers targeting receptors present on TAMs were conducted in preclinical animal models (Table 1), whereas just a few tracers were tested in patients. The majority of PET imaging studies targeting receptors present on TAMs were performed in animal models for (neuro)inflammatory diseases, rather than tumor models. Nonetheless, these studies can provide insight into the binding properties of ligands to receptors or enzymes of interest, which can be translated to studies with tumor models or cancer patients.

**Table 1:** Summary of potential PET tracers for TAMs, evaluated in preclinical or clinical studies

Target	Macrophage phenotype	Radiotracer	Species	Disease (model)	References
CD206	M2	[^18^F]FDM	Rabbit	Atherosclerosis model	Tahara et al. (2014)
		[^18^F]FB-anti-MMR-sdAb	Mouse	Lung cancer xenograft model	Blykers et al. (2015)
		[^68^Ga]NOTA-MSA	Mouse	Atherosclerosis model	Kim et al. (2016)
		[^68^Ga]NOTA-anti-MMR-Nb	Mouse	Atherosclerosis model	Varasteh et al. (2019)
		[^68^Ga]Ga-NOTA-anti-MMR-sdAb	Mouse	Lung cancer xenograft model	Xavier et al. (2019)
CD163	M2	[^68^Ga]ED2	Rat	Arthritis model	Eichendorff et al. (2015)
Arginase	M2	[^18^F]FMARS	Mouse	Prostate cancer xenograft model	Clemente et al. (2021)
		[^18^F]FBMARS	Mouse	Prostate cancer xenograft model	Clemente et al. (2021)
CSF-1R	M1 and M2	[^11^C]AZ683	Rat, non-human primate	Healthy	Tanzey et al. (2018)
		[^11^C]CPPC	Mouse, non-human primate	Neuroinflammation model (LPS)	Horti et al. (2019), Zhou et al. (2021)
		[^11^C]GW2580	Mouse, non-human primate	Neuroinflammation mouse model (LPS) and health non-human primates	Zhou et al. (2021)
CD68	M1 and M2	[^64^Cu]CD68-Fc	Mouse	Atherosclerosis model	Bigalke et al. (2014)
CCR2	M1 and M2	[^64^Cu]-DOTA-ECLi	Rat	Abdominal aortic aneurysm model	English et al. (2020)
		[^64^Cu]Cu@CuOx-ECLi	Mouse	Pancreatic ductal adenocarcinoma model	Zhang et al. (2021)
		[^18^F]6b	Mouse	Healthy	Wagner et al. (2021)
Folate receptor	M1 and M2	[^68^Ga]NOTA-folate	Mouse	KB xenograft model	Brand et al. (2017)
		[^18^F]AlF-NOTA-folate-PEG_12_	Mouse	KB xenograft model	Chen et al. (2017)
		[^18^F]fluoro-PEG-folate	Mouse	Rheumatoid arthritis model	Chandrupatla et al. (2018)
			Human	Rheumatoid arthritis	Verweij et al. (2020)
		[^18^F]AlF-NOTA-folate	Mouse	Atherosclerosis model	Silvola et al. (2018)
		[^18^F]AzaFol	Mouse	Lung fibrosis model	Schniering et al. (2019)
			Human	Ovarian and lung cancer	(https://clinicaltrials.gov/ct2/show/NCT03242993)

sdAb: single-domain antibody; Nb: nanobody; CSF-1R: Colony stimulating factor 1 receptor; CD: cluster of differentiation; CCR2: C–C chemokine receptor type 2**;** KB: human epithelial cancer cell; Fc: fragment crystallizable region; LPS: lipopolysaccharides; 6b: 2-[4-(5-Fluoropentoxy)phenyl]-N-{4-[N-methyl-N-(tetrahydro-2H-pyran-4-yl)aminomethyl]phenyl}-6,7-dihydro-5H-benzo[7]annulene-8-carboxamide

### Targets mainly expressed by TAMs

#### CSF-1R

Colony Stimulating Factor 1 receptor (CSF-1R) is a member of the tyrosine kinase receptor family, mainly expressed on macrophages (Zhang et al. 2020). The CSF-1R plays an important role in the macrophage’s proliferation and differentiation, which can be stimulated by tumor cells via CSF1 secretion (CSF-1R ligand) (Fischer et al. 2009).^.^ The CSF-1R seems to be a potential target for imaging of macrophages/microglia, since this receptor is highly expressed on these cells in the TME and at sites of inflammation (Cendrowicz et al. 2021). Several studies have shown that the CSF-1R can be expressed by macrophages of both the M1 and M2 phenotype (Fischer et al. 2009; Cannarile et al. 2017), although it is still inconclusive in what phenotype it is predominantly expressed. High expression of CSF-1R in tumors has been related to poor prognosis.

**Fig. 2:**
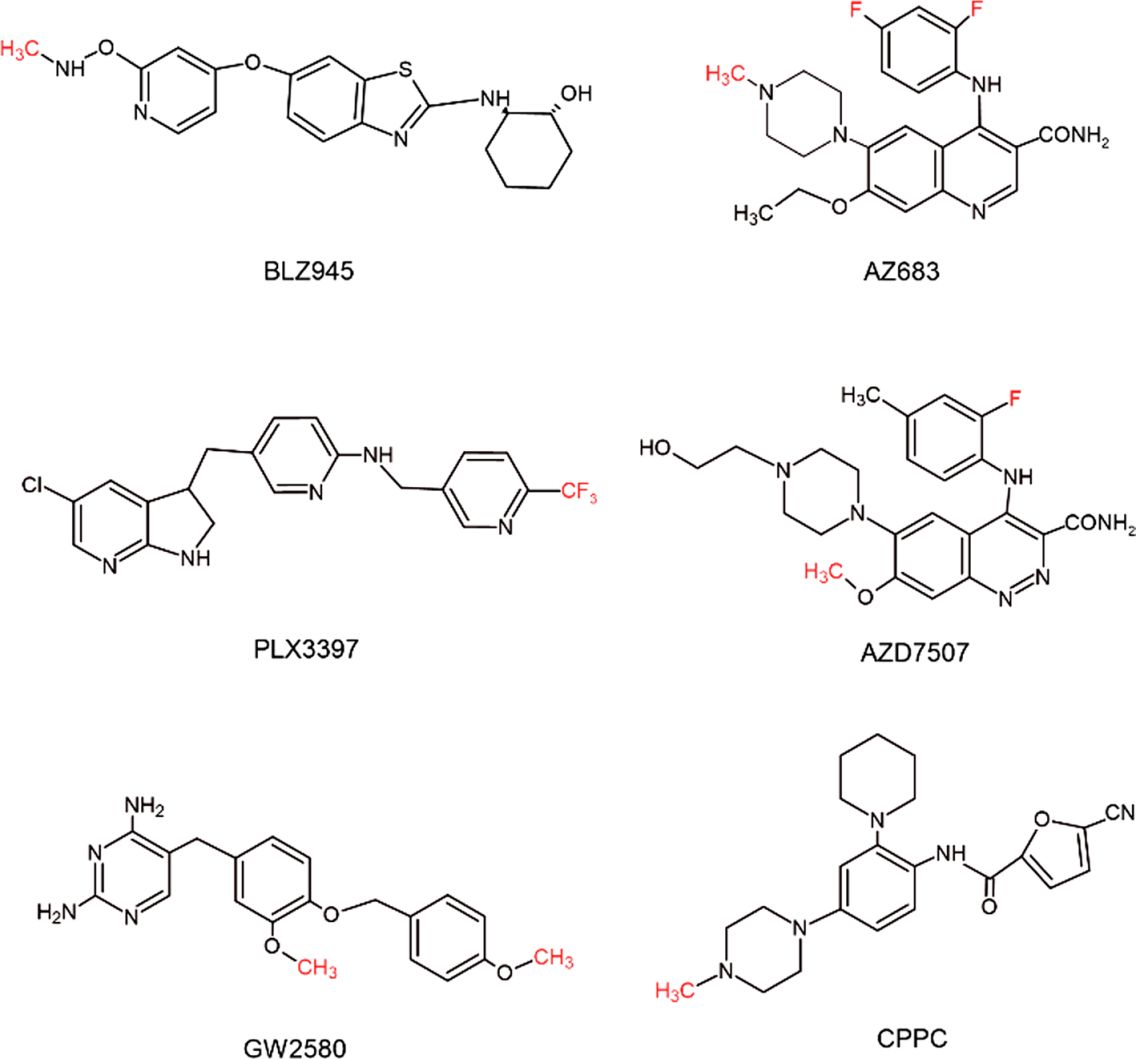
Structures of representative receptor ligands of the CSF-1R. The positions of the CSF-1R ligands that have been radiolabeled and evaluated as candidate PET tracers are highlighted in red. CSF-1R: colony stimulating factor 1 receptor

Several studies have reported inhibition of the CSF-1R as a potential strategy for TAM depletion (Noy and Pollard 2014; Valero et al. 2021). For instance, the CSF-1R inhibitor PLX3397 was evaluated in a phase 1 clinical trial in patients with tenosynovial giant-cells tumors. After 4 months of treatment, 11 of 14 patients had a mean decrease in tumor volume score of 61%. The remaining 3 patients had stable disease (Tap et al. 2015). In a preclinical study conducted by Pyonteck et al., the compound BLZ945 improved long-term survival in a glioblastoma mice model, with 64.3% of the mice surviving up to the 26 weeks end-point (Pyonteck et al. 2013). Therefore, imaging the CSF-1R expression on macrophages might help the physician to select patients for CSF-1R targeting therapy, and monitoring of the patient therapy response. Besides these applications, PET imaging of CSF-1R could also be useful for the evaluation of new specific and sensitive CSF-1R inhibitors that have been emerged as potential TAM-therapies, as well as a better understanding of CSF-1R mechanisms involved in the tumor progression (Cendrowicz et al. 2021; Zhang et al. 2020; Noy and Pollard 2014). Several CSF-1R ligands, including AZ683, BL2945, PLX3397, GW2580, AZD7507, and CPPC, have been radiolabeled and tested as candidate PET tracers for CSF-1R (Fig. 2) (Tanzey et al. 2018; Horti et al. 2019). Tanzey et al. used [^11^C]AZ683 for imaging the brain of healthy rats and non-human primates (Tanzey et al. 2018). Even though the tracer seemed to be a good candidate due to its high target selectivity, low plasma protein binding and suitability to be labeled with carbon-11 and fluorine-18, [^11^C]AZ683 when evaluated in vivo showed little brain uptake and high uptake in pituitary and thyroid glands. These findings might indicate either non-specific binding or that [^11^C]AZ683 might be a possible substrate for the P-glycoprotein transporter. Therefore, the authors suggested to use this tracer in future studies in peripheric inflammation or tumor models, but no reports of such studies have been published so far.

Horti et al. conducted the first study demonstrating the selectivity of [^11^C]CPPC for the CSF-1R on microglia in pre-clinical studies (Horti et al. 2019). The radiotracer is lipophilic enough to pass through the BBB (Log D_7.4_ = 1.6) and has a similar IC_50_ (0.8 nM) as other CSF-1R ligands (e.g. BLZ945 1.2 nM; and PLX3397 20 nM). Studies conducted in animal models of neuroinflammation (LPS-induced) and neurodegenerative diseases (Alzheimer’s diseases; Multiple Sclerosis) indicate the tracer binds specifically to CSF-1R. *Post-mortem* investigations on human brain slices also show CSF-1R mediated specific binding (Valero et al. 2021). Recently, Zhou et al. developed GW2580 (IC_50_ = 10 nM) labeled with ^11^C and compared this tracer to [^11^C]CPPC in a rodent model for neuroinflammation and in non-human primates (Tanzey et al. 2018). In the mice inflammation model, [^11^C]GW2580 showed a 1.8-fold higher uptake than [^11^C]CPPC, suggesting slightly higher sensitivity of the former tracer. Blocking studies demonstrated approximately 30% less uptake of [^11^C]GW2580 in the normal non-human primate brain after administration of unlabeled GW2580 than at baseline. On the other hand, minimal blockade of [^11^C]CPPC uptake after administration of unlabeled CPPC was observed under normal physiological conditions. Therefore, Zhou et al. suggested that [^11^C]GW2580 might be a superior candidate tracer for imaging of CSF-1R.

None of the radiopharmaceuticals described in this section were tested in tumor models or in humans. Based on brain imaging studies in animals, [^11^C]GW2580 seems to be the most suitable PET tracer for CSF-1R, as it showed higher specific brain uptake than [^11^C]AZ683 and [^11^C]CPPC.

### Scavenger receptors

The scavenger receptor family (SR-A, SR-B, SR-C, SR-D, SR-E, SR-F, SR-G, SR-H, SR-I, SR-J, SR-K, SR-L) plays a vital role in the clearance of endogenous and host molecules from the TME (PrabhuDas et al. 2017). The SR-D1, also known as CD68, is the only member of this family that can be found on monocytes and macrophages. CD68 is a transmembrane protein that participates in oxidized low-density protein (OxLDL) clearance (e.g., lipid-laden foam cells in atherosclerosis) (PrabhuDas et al. 2017) and is considered a pan-macrophage marker (PrabhuDas et al. 2017; Yang et al. 2019; Jeong et al. 2019). High expression of the CD68 marker in tumors, such as in breast, cervix, and bladder carcinoma, has been correlated with a poor prognosis (Jeong et al. 2019). Bigalke et al. labeled a CD68 antibody fragment (Fc) with Cu-64 ([^64^Cu]CD68-Fc) to detect foam cells (fat-laden M2 macrophages containing low-density lipoproteins) in an atherosclerosis model (Bigalke et al. 2014). The ApoE−/− mice that received a fat-rich diet showed a higher tracer uptake in the aortic arch when compared to wild-type control mice. This finding correlated well with the results from magnetic resonance imaging (R1-mapping) and ex vivo analyses, such as histology and immunostaining (Bigalke et al. 2014). Although the [^64^Cu]CD68-Fc was used to detect foam cells in cardiovascular diseases, this tracer may also be useful for imaging of TAM.

**Fig. 3:**
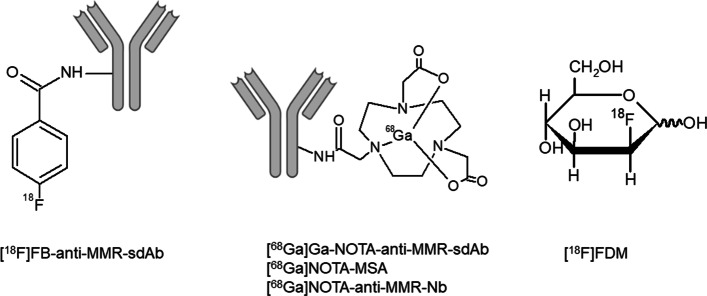
Structures of representative candidate tracers for the CD206 receptor

The macrophage mannose receptor (MMR, SR-E3 subtype), also known as CD206, might be a suitable M2-specific marker (Blykers et al. 2015; PrabhuDas et al. 2017). CD206 is a C-type lectin transmembrane protein able to detect and phagocytose pathogens (PrabhuDas et al. 2017). Radiotracers for CD206 (Fig. 3), such as [^18^F]FDM, [^68^Ga]NOTA-MSA, and [^68^Ga]NOTA-anti-MMR-nanobody (Nb), have been developed and studied mainly in animal models for atherosclerosis (Tahara et al. 2014; Blykers et al. 2015; Kim et al. 2016; Varasteh et al. 2019; Xavier et al. 2019). A few tracers have been tested in preclinical tumor models, in particular [^18^F]FB-anti-MMR-single-domain antibody (sdAb) and [^68^Ga]Ga-NOTA-anti-MMR-sdAb (Blykers et al. 2015; Xavier et al. 2019). *Xavier *et al*.* demonstrated that [^68^Ga]Ga-NOTA-anti-MMR-sdAb showed higher uptake in tumors, liver and spleen (organs with a high density of M2 macrophages) of wild-type tumor-bearing mice (Lewis lung carcinoma model) as compared to CD206 knock-out mice (Xavier et al. 2019). The wild-type group showed 3.54 times higher [^68^Ga]Ga-NOTA-anti-MMR-sdAb uptake than the knock-out mice when expressed as tumor-to-blood ratio. Likewise, *Blykers *et al*.* observed that [^18^F]FB-anti-MMR-sdAb showed significantly (*ca.* eightfold) lower uptake in the tumors of knock-out mice when compared to wild-type mice (Blykers et al. 2015), suggesting that the tracer binds specifically to CD206. Besides the high tumor uptake found for the anti-MMR-sdAb labeled with gallium-68 or fluorine-18, the tracers also presented high specific uptake in organs expressing MMR, such as liver, spleen, lymph nodes, and bone marrow (Blykers et al. 2015; Xavier et al. 2019). Despite [^18^F]FB-anti-MMR-sdAb shows to be a potential PET tracer for CD206 receptor, dosimetry and toxicity studies still are warranted for tracer evaluation. Therefore, *Xavier *et al*.* suggested [^68^Ga]Ga-NOTA-anti-MMR-sdAb for future evaluation in clinical studies, since this tracer was already demonstrated to have safe dosimetry and its labeling can easily be implemented in radiopharmacies with a ^68^Ge/^68^Ga generator.

The cysteine-rich scavenger receptors (SRCR; SR-I family), such as CD163, have been extensively studied in order to understand the M2 macrophage phenotype (Graversen and Moestrup 2015). CD163 is a transmembrane protein responsible for haptoglobin-hemoglobin (Hp-Hb) clearance. It is considered an anti-inflammatory receptor due to its capacity to clear free Hb and to promote the production of anti-inflammatory mediators (Skytthe et al. 2020; Yang et al. 2019). This receptor seems to be a good target for imaging of M2-like macrophages, since several studies have reported a high density of CD163+ macrophages in the TME, which correlated with a poor prognosis (Skytthe et al. 2020; Li et al. 2021). Until now, the tracers developed for this receptor were evaluated in arthritis and atherosclerosis animal models. *Eichendorff *et al*.* labeled a CD163 antibody with gallium-68 ([^68^Ga]ED2) and showed highly selective binding to transfected CHO cells overexpressing the rat CD163 receptor. In vivo studies in a collagen-induced arthritis (CIA) rat model showed higher liver and lower spleen uptake of [^68^Ga]ED2 in the CIA group, when compared to healthy animals (Eichendorff et al. 2015). This might be explained by the immune response induced by CIA with higher infiltration of monocytes possibly derived from the spleen. Spleen contains a storage of monocytes that can be released into the circulation in peripheral inflammatory response (Eichendorff et al. 2015). The CIA model promotes an immunological response causing modulation of M2 polarization in the liver (Kupffer cells), spleen (splenic red pulp) and the paw. Consequently, CIA caused a 1.83-fold increase in [^68^Ga]ED2 uptake in the inflamed paw compared to the health paw. Yet, the [^68^Ga]ED2 uptake in the liver and spleen was much higher than in the inflamed paw. *Eichendorff *et al*.* hypothesized that this difference in uptake might be due to ineffective antibody penetration in the inflamed tissues due to its size. Another possible explanation is the fact that the biological half-life of large biomolecules such as antibodies (days to weeks) does not match with the physical half-life of the gallium-68 (68 min) (Tsai and Wu 2018), which precluded delayed image acquisition. The use of an antibody fragment (Fab) or small molecules like peptides instead of the entire anti-CD163 antibody might increase tissue penetration and decrease the biological half-life, allowing the radiolabeling with gallium-68. *Silva *et al*.* conducted a study using a novel peptide, CTHRSSVVC, labeled with indium-111 ([^111^In]DOTA-CTHRSSVVC) and showed high tracer uptake in the atheroma of low-density protein receptor-deficient mice ex vivo (Silva et al. 2016). Besides being a promising candidate for CD163 receptor imaging with single-photon emission computerized tomography (SPECT), the peptide could be labeled with a positron-emitting isotope and used for PET imaging, which offers a higher sensitivity and resolution and better quantification options than SPECT.

In summary, [^64^Cu]CD68-Fc seems to be a good candidate tracer for CD68, which could be used for general TAM imaging. However, this tracer has not been tested in tumor models yet. On the other hand, [^68^Ga]Ga-NOTA-anti-MMR-sdAb seems to be a suitable PET tracer for imaging of TAM of the M2-phenotype. This tracer was successfully tested in tumor models and presented good outcomes in dosimetry studies. Although the CD163 receptor also seems to be a good target for imaging of macrophages of the M2-phenotype, no suitable PET tracer for this target is available yet. None of the PET tracers for scavenger receptors has been evaluated in patients yet.

### CCL2/CCR2

Chemokine (C–C Motif) ligand 2 (CCL2), also known as monocyte chemo-attractant protein-1 (MCP-1), is responsible for the recruitment of immune cells to the site of inflammation. However, when its response is exacerbated, it can lead to the development of several pathologies (Baggiolini 1998). In the case of tumors, the exacerbated recruitment of immune cells, especially of activated monocytes and immunosuppressive TAMs, can lead to the formation of an immune cell network in the TME that leads to tumor progression (Hao et al. 2020). CCL2 can also be found in the blood stream and has been proposed as biomarker for cancer diagnosis (Tsaur et al. 2015; Lubowicka et al. 2018). *Deci *et al*.* demonstrated that chemokine receptor type 2 (CCR2) inhibition with the single-chain variable fragment 58C-scFv (selected by phage display) repolarizes macrophages from M1 to M2, suggesting that CCR2 might be linked to the M2 phenotype (Deci et al. 2018). New therapeutic inhibitors of CCR2, such as carlumab and PF-04136309, have been used in clinical trials to block CCL2/CCR2 signaling, leading to inhibition of TAM infiltration, and consequently, reduction of tumor progression (Zhang et al. 2020).

**Fig. 4:**
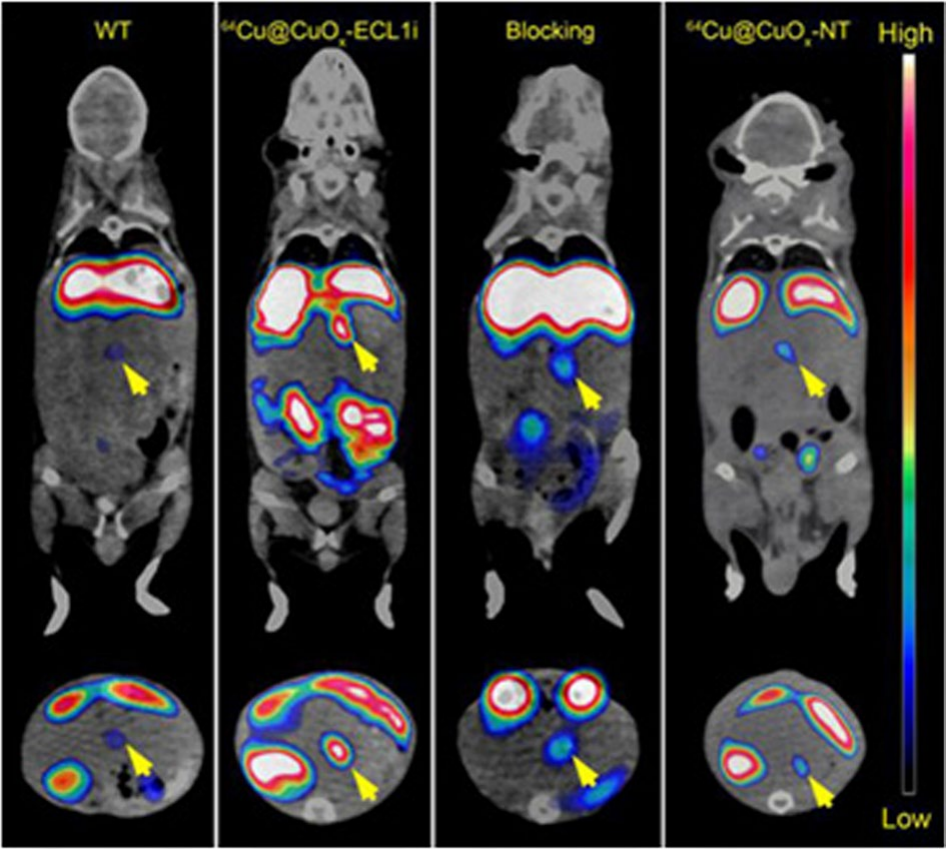
Representative in vivo PET/CT images of [^64^Cu]Cu@CuOx-ECL1i. Images of [^64^Cu]Cu@CuOx-ECL1i in KPPC mice, KPPC mice with 50-fold blocking dose, and [^64^Cu]Cu@CuOx-NT in KPPC mice were performed at 24 h post injection (yellow arrow: pancreas/pancreatic tumor). Reprinted (adapted) with permission from X. Zang et al. ACS Nano 2021 (Zhang et al. 2021). Copyright © 2021 American Chemical Society

Recently, PET tracers were developed for imaging of the CCR2. *Wagner *et al*.* developed the [^18^F]2-[4-(5-fluoropentoxy)phenyl]-N-{4-[N-methyl-N-(tetrahydro-2H-pyran-4-yl)aminomethyl]phenyl}-6,7-dihydro-5H-benzo[7]annulene-8-carboxamide, a ^18^F-labeled CCR2 selective antagonist (called [^18^F]6b), that showed high binding affinity to the CCR2. The tracer was stable in vitro and in vivo, and showed little defluorination and a fast uptake in the liver, spleen, lungs and kidneys of healthy mice. However, preclinical evaluation of this tracer in a pathological model has not been reported so far (Wagner et al. 2021). Another group labeled the peptide ECLi with copper-64 and used it to assess the expression of CCR2 in a rodent model of an abdominal aortic aneurysm (AAA), in which monocytes and macrophages play a critical role. The authors reported a high sensitivity and specificity for detecting CCR2^+^ cells in the AAA model. Additionally, [^64^Cu]DOTA-ECLi also demonstrated specific binding to ex vivo human AAA specimens with high expression of CCR2+ cells (English et al. 2020). So far, only a few studies explored the potential of PET imaging of CCR2-expressing macrophages in tumor models. *Zhang *et al*.* used ultra-small radiolabeled copper nanoparticles, [^64^Cu]Cu@CuOx, coupled with the ECLi peptide in an animal model of pancreatic ductal adenocarcinoma (PDAC) (Zhang et al. 2021). Target specificity and sensitivity was evaluated in vitro and in vivo. Firstly, PDAC cells derived from mice (KI) and human monocyte THP-1 cells were used to assess the tracer uptake. In both KI and THP-1 cells, [^64^Cu]Cu@CuOx-ELCi uptake was nearly fivefold higher than uptake of the non-targeted analogue [^64^Cu]Cu@CuOx. Competition with non-labeled Cu@CuOx-ELCi as blocking agent resulted in an over 80% reduction in the [^64^Cu]Cu@CuOx uptake in both cell lines. In the PDAC mice model, [^64^Cu]Cu@CuOx-ELCi demonstrated a threefold higher uptake than the non-targeted [^64^Cu]Cu@CuOx analogue (Fig. 4) (Zhang et al. 2021).

Overall, both PET tracers based on the ELCi molecule demonstrated specific binding to CCR2, but only [^64^Cu]Cu@CuOx-ELCi was tested in tumor-bearing mice. No studies with these tracers have been performed in humans. Despite the early stage in tracer development, these tracers could be a suitable candidate PET tracer for general TAM imaging, provided that the remaining qualification of the tracers is successful.

### Folate receptors

Folate receptors (FR) are *N*-glycosylated proteins that can have different isoforms (FRα, FRβ, FRγ, and FRδ), which are overexpressed in different cell types. FRα can be found in epithelial cells and certain cancers with epithelial origin, such as ovary, breast, bladder, epithelium, and colon (Shen et al. 2018; Sosnik 2018). FRβ is expressed by monocytes and activated macrophages (Brand et al. 2017; Kurahara et al. 2012; Puig-Kröger et al. 2009). Recently, high expression of FRβ was observed in IL-10 and CD163-expressing macrophages of the M2 phenotype (Choi et al. 2018; Newman and Maddocks 2017). FRβ expressing macrophages are not only present in the TME but also in other inflammatory diseases, such as rheumatoid arthritis (Shen et al. 2018; Kurahara et al. 2012; Choi et al. 2018). Little information is available regarding the FRγ and FRδ subtypes, but they can be found on regulatory T cells and at low concentrations in blood, respectively (Shen et al. 2018).

Folic acid (FA) binds with high affinity to all folate receptors and is internalized by endocytosis. Folic acid is converted to tetrahydrofolate (THF) by dihydrofolate reductase (DHFR) and this step is an important target for antifolate drugs, such as methotrexate, pemetrexed, and raltitrexed (Newman and Maddocks 2017). THF is essential for one-carbon supply to the purine, methionine and thymidine synthesis, and consequently the proliferation of cells. Cancer cells can use this pathway for survival and proliferation (Newman and Maddocks 2017). Therefore, FA and FA-derivatives have been considered a potential drugs in cancer research (Brand et al. 2017; Silvola et al. 2018; Choi et al. 2018; Boss et al. 2018).

**Fig. 5:**
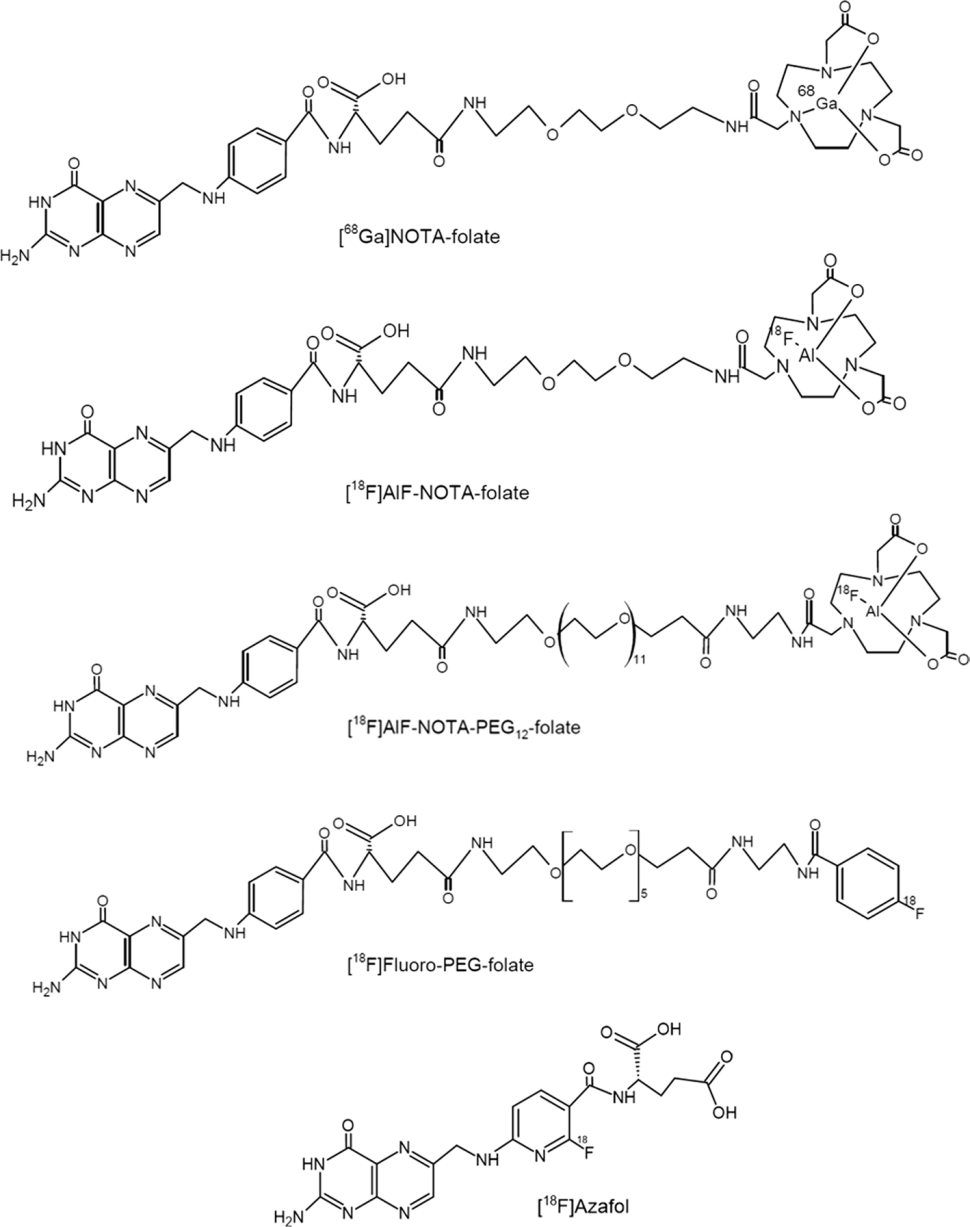
Structures of representative candidate tracers for FRβ. FRβ: folate receptor β

In addition to its applications in cancer therapy, imaging agents based on FA-derivatives have been developed during the last years (Fig. 5). *Silvola *et al*.* synthesized the [^18^F]AlF-NOTA-folate, which presented good in vivo stability (85%) and significant specific binding to the FR in the plaques in an atherosclerotic rabbit model (*ca.* 82%) and in an atherosclerotic mouse model (*ca.* 92%), as measured ex vivo with autoradiography (Silvola et al. 2018). Binding studies with [^18^F]AlF-NOTA-folate on human carotid endarterectomy samples stained positive for FRβ and CD68 also showed significant blocking (*ca.* 88%) of tracer binding, when the folate glucosamine was co-administered. Although specific binding of [^18^F]AlF-NOTA-folate to FR on the polarized M2 macrophage phenotype was observed in in vitro studies, specific binding to the M1 macrophage phenotype was demonstrated in atherosclerotic mice. Therefore, [^18^F]AlF-NOTA-folate might be a potential tracer for FRβ imaging on both macrophage phenotypes (Silvola et al. 2018). Another study conducted by *Schniering *et al. demonstrated a good correlation between the uptake of the tracer [^18^F]AzaFol and FRβ expression by macrophages in a lung fibrosis mice model (Schniering et al. 2019). Blocking studies using folic acid showed a significantly reduction in [^18^F]AzaFol uptake in the fibrotic lung. However, all imaging studies were based on ex vivo analysis and static image acquisition. Therefore, the authors suggest that a dynamic PET scan should be performed in the following experiments where correction for changes in the pulmonary blood flow could be done to avoid the possible non-specific accumulation of [^18^F]AzaFol in the pulmonary disease model. Despite the limitations presented in this study, [^18^F]AzaFol is currently being evaluated in early-phase clinical studies in patients with ovarian or lung cancer (https://clinicaltrials.gov/ct2/show/NCT03242993). The first-in-man dosimetry studies of [^18^F]AzaFol demonstrated suitable dosimetry outcomes for six patients presenting lung adenocarcinoma, resulting in an estimated effective dose of 18.0 ± 2.6 μSv/MBq. The highest absorbed doses found were in the liver, kidneys, bladder, and spleen (51.9 ± 16.4, 45.8 ± 8.3, 39.1 ± 16.8, and 35.4 ± 39.7 μGy/MBq, respectively) whereas tumor presented an average of 34.8 ± 17 μGy/MBq. Although favorable dosimetry outcomes were observed, this study was conducted with only few lung cancer patients. Therefore, further investigation using [^18^F]AzaFol is warranted to confirm these results.

Other folate-based tracers, such as [^68^Ga]NOTA-folate and [^18^F]AlF-NOTA-folate-PEG_12_, were recently developed and presented satisfactory results in a human epidermal carcinoma xenograft mouse model, with low accumulation in the liver and high target specificity (Brand et al. 2017; Chen et al. 2017). *Chen *et al*.* reported similar biodistribution results for [^18^F]AlF-NOTA-folate-PEG_12_ and the clinically applied SPECT imaging agent [^99m^Tc]EC20 (Chen et al. 2017). The pharmacokinetic profile of [^18^F]Al-NOTA-PEG_12_-folate was improved by raising its hydrophilicity, leading to a lower liver uptake, less background and consequently a better target-to-background contrast for detection of hepatic cancer (Chen et al. 2017). In another study conducted by *Brand *et al*.*, similar biodistribution results between [^68^Ga]NOTA-folate and [^99m^Tc]EC20 were observed. However, [^68^Ga]NOTA-folate had a lower liver uptake and faster blood clearance than [^99m^Tc]EC20, making it a promising candidate for further evaluation (Brand et al. 2017). *Chandrupatla *et al*.* used [^18^F]fluoro-PEG-folate for the detection of systemic inflammation in the liver and spleen of arthritic rats. The tracer was also used for methotrexate treatment monitoring. After the anti-inflammatory treatment, a twofold lower uptake was observed in the liver and spleen of arthritic rats. The authors attribute these results to the reduction of activated macrophages as a result of the drug therapy (Chandrupatla et al. 2018). In addition, [^18^F]fluoro-PEG-folate showed fast tracer uptake (~ 1 min) in inflamed joints and fast washout from blood of patients presenting rheumatoid arthritis in the first in man study (Verweij et al. 2020). [^18^F]fluoro-PEG-folate demonstrated significant higher target-to background uptake ratio in joints (3.5 ± 2.2) when compared to [^11^C]PK11195 (1.7 ± 0.6). However, the study comparing these tracers was not performed in the same patients and therefore, a head to head comparison was not possible which may interfere in the true tracers comparison.

Overall, folate-based PET tracers might be a good approach for general TAM imaging since folate receptors are expressed by both macrophage phenotypes. Among the tracers described in this section, [^18^F]AzaFol is the only tracer that is under evaluation in humans presenting cancer.

### Arginase

Arginase is an enzyme that catalyzes the hydrolysis of L-arginine to L-ornithine and urea. It is present in two isoforms: type I (Arg1), which is mainly expressed in the liver, and type II (Arg2), which is expressed in virtually all tissues. Arg1 is involved in the production of urea for ammonia clearance and in the synthesis of L-ornithine. Arg2 regulates the synthesis of L-ornithine in other tissues (Gonçalo et al. 2020; Grzywa et al. 2020). Arginase levels are negatively correlated with the activity of neuronal, endothelial and inducible nitric oxide synthases (nNOS/eNOS/iNOS) (Gonçalo et al. 2020; Rath et al. 2014; Thomas and Mattila 2014). L-arginine is a precursor for the production of nitric oxide (NO) and consequently arginase and NO synthases compete for the same substrate (Gonçalo et al. 2020; Rath et al. 2014; Thomas and Mattila 2014). Oxidative and inflammatory signaling pathways may disturb the arginase/NO physiological equilibrium. Overexpression of arginase results in a decrease in NO levels and an increase in proline and polyamine levels, which have been related with cardiovascular, inflammatory and immune-mediated pathologies. More importantly, arginase is upregulated mainly by myeloid cells in the TME at the early stages of tumor development and it is associated with poor outcomes. In addition to being expressed by tumor-infiltrating cells, arginase is also known to be expressed in some tumor cells (Grzywa et al. 2020). Arginase overexpression stimulates tumor cell proliferation and evasion from the immune system. Inhibition of arginase would direct L-arginine metabolism towards the NO pathway, promoting inhibition of tumor response, infiltration of M1-like macrophages and the repolarization of M2-like to M1-like macrophages in the TME, which results in immune stimulation and consequently anti-tumor activity (Gonçalo et al. 2020; Grzywa et al. 2020). Thus, inhibition of immunosuppressive functions of arginases has been explored in the treatment of cancer using arginase inhibitors.

**Fig. 6:**
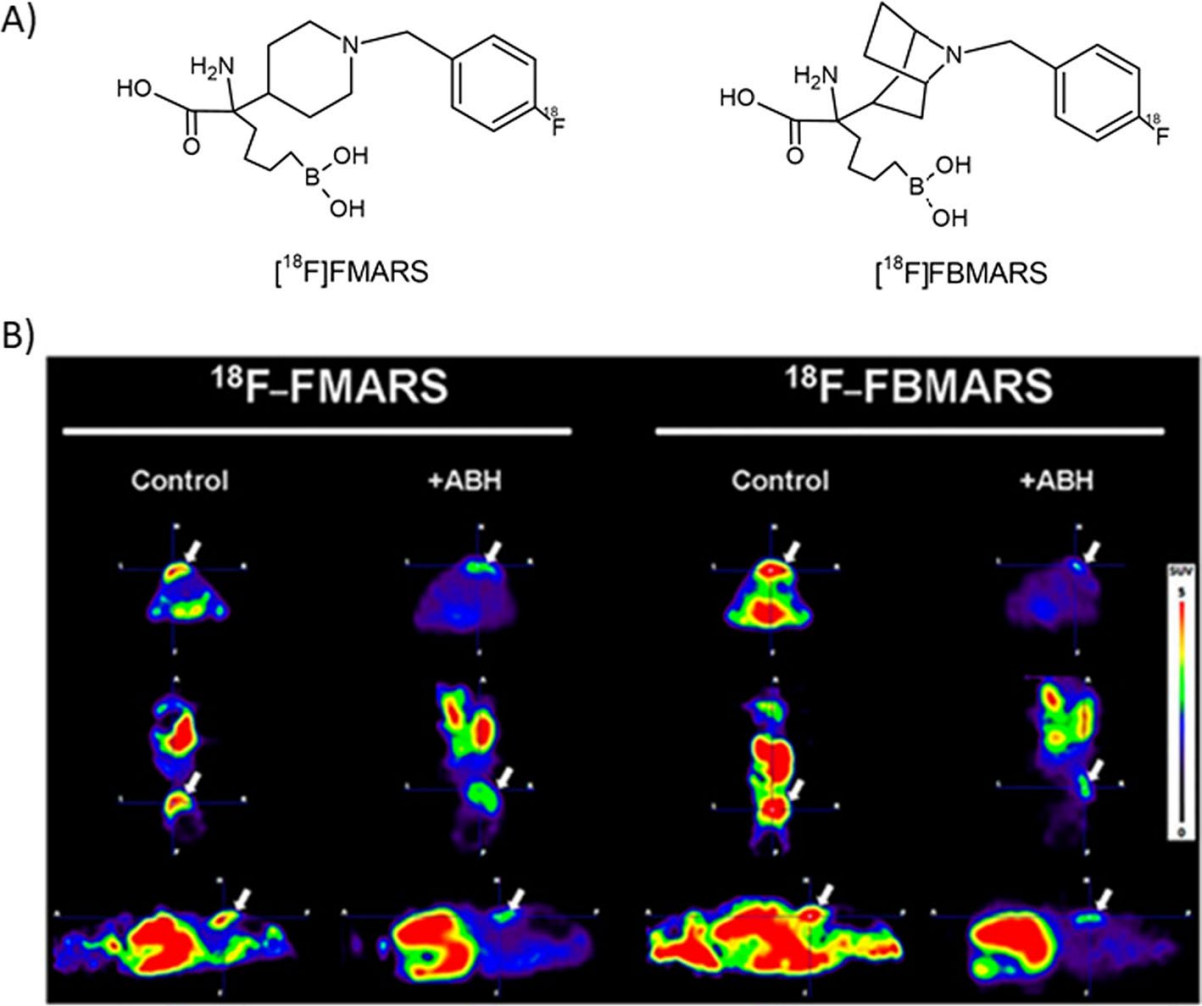
Structures (**a**) and PET images (**b**) of representative candidate tracers for arginase. PET images at 40–90 min post injections of [^18^F]FMARS and [^18^F]FBMARS in PC3 tumor (arrows; axial, coronal, and sagittal views from top to bottom) of mice without (control) and with coinjection of ABH (5 mM). ABH: (2(S)-amino-6-boronohexanoic acid. This research was originally published in *JNM.* G.S. Clemente, et al. J. Nucl. Med. 2021 (Clemente et al. 2021). Copyright © 2021 by the Society of Nuclear Medicine and Molecular Imaging

Arginase inhibitor-based tracers have not been extensively explored yet. Recently, our research group developed two PET tracers targeting arginase, [^18^F]FMARS and [^18^F]FBMARS (Fig. 6) (Clemente et al. 2021). PET imaging and biodistribution studies in mice bearing a PC3-xenograft demonstrated arginase-mediated uptake. PET scans of the xenograft animal model demonstrated a high tracer uptake in PC3 tumors. Tracer uptake was significantly reduced by *ca.* 60% when (2(S)-amino-6-boronohexanoic acid (ABH, an arginase inhibitor) was co-injected, which confirms tracer specificity. Since [^18^F]FBMARS displayed almost twofold higher tumor uptake than [^18^F]FMARS (Fig. 6), with a significantly blocking (*ca.* 70%) by ABH, and presented tumor-to-organ ratios higher than two in ex vivo biodistribution studies, [^18^F]FBMARS was suggested to be the more suitable tracer to further be evaluated towards application in humans (Clemente et al. 2021).

## Conclusions

Immunotherapies targeting TAMs seem to be an attractive strategy to decrease tumor progression. Several drugs have emerged in the last decade that could be applied to those patients that usually do not respond to common therapies. The assessment of TAM phenotype is important for patient stratification. Molecular imaging techniques to assess the expression of specific receptors by the TAM have been used to better characterize the tumor profile. PET imaging of the tumor with specific ligands for biomarkers of TAM might help to predict and monitor the response to TAM-targeting therapies.

Several biomarkers on TAM have been already evaluated as target for the assessment of TAMs with PET (Table 1). Several biomarkers presented in this review are expressed by both the M1 and the M2 macrophage phenotypes, in particular CSF-1R, CD68, CCR2, and FRβ. Although these markers are not phenotype selective, they still remain important targets to evaluate the total TAM population in the TME. These macrophage markers are highly expressed in several types of cancer and have been investigated as potential targets for treatment with specific inhibitors (Zhou et al. 2020; Lubowicka et al. 2018; Kurahara et al. 2012; Jamiyan et al. 2020). So far, PET tracers, such as [^11^C]GW2580 (CSF-1R), [^64^Cu]Cu@CuOx-ELCi (CCR2), and [^18^F]AzaFol (folate receptor), have shown potential as radiopharmaceuticals for general TAM assessment. Although high infiltration of macrophages in the TME is correlated to a poor patient prognosis (Poh and Ernst 2018; Chen et al. 2019), the phenotype is important for the immunotherapeutic approach to be selected. As mentioned previously, the M1-like phenotype is related to a better prognosis and the M2-like phenotype to a worse prognosis (Ngambenjawong et al. 2017), making the latter a potential target for tumor regression. So far, PET tracers have been developed for only three targets that are specifically expressed by macrophages of the M2 phenotype, in particular CD206, CD163, and arginase, whereas no PET tracers for targets specifically expressed by M1-like macrophages were found. Among the M2 specific makers reported, the CD206 has already been considered a specific marker for M2 phenotype for many years. Recently, arginase was also proposed to be a potential M2-selective marker, since it is highly expressed by the M2 macrophage phenotype in tumors and does not present expression by M1 macrophages (Cassetta and Pollard 2018). It is important to highlight that M2-type macrophages are also present to some extent under normal conditions (Kambara et al. 2015; Veremeyko et al. 2018; Qi et al. 2016), which could give rise to a small basal, non-disease related signal. A few potential PET tracers for CD206 have demonstrated suitable properties that warrant further evaluation. Among them, [^68^Ga]Ga-NOTA-anti-MMR-Sdab (CD206 receptor) can be highlighted due to the demonstrated good selectivity and safe dosimetry in animals, and therefore, may be a step closer to be available for the clinical trials comparing to others. In addition, [^18^F]FBMARS presented high specificity for arginase in a tumor-bearing mouse model. However, despite being presented as a potential target to assess M2-macrophages, the arginase expression might be also stimulated by tumor cells and therefore, [^18^F]FBMARS does not present selectivity for M2-macrophages tracking (Thomas and Mattila 2014). In contrast, the increased arginase expression provided by both cells above-mentioned in the TME might lead [^18^F]FBMARS to become a suitable PET tracer for arginase inhibitor therapies.

Additionally, despite CD163 receptor being considered a selective marker for M2-type macrophages, no suitable PET tracer has been developed until now. Likewise, no specific PET tracer for the M1-TAM phenotype was developed so far. This observation might be due to the fact that most markers on M1-type macrophages (e.g. the CD80 and CD86 receptor) are not specific for macrophages, as they are also expressed by other cells, such as antigen-presenting cells (e.g. dendritic cells, and B cells) [82]. Another explanation is the higher interest of researchers for the M2-phenotype, since it is instrumental in the worse patient prognosis and thus a more attractive drug target.

Interestingly, the majority of specific PET tracers for TAMs were only evaluated preclinically (Table 1), except for the two folate tracers [^18^F]AzaFol, which is a general TAM tracer that is being evaluated in lung and ovarian cancer in clinical trials, and [^18^F]fluoro-PEG-folate folate that was tested in first in man study with patients presenting rheumatoid arthritis. The translation from preclinical to clinical studies is a lengthy process that may be influenced by several factors, including the fact that preclinical models are not able to fully mimic human disease [83]. In addition, radiotracers in early preclinical development usually fail because they do not present enough specificity or stability in vivo. Nonetheless, TAM targets need to be further explored, and the already existing PET tracers further tested in tumor models and subsequently in patients.

To summarize, imaging TAMs using PET tracers is still in its infancy, but seems to be a potential approach for early tumor characterization, response prediction and treatment monitoring, mainly in patients who present tumor-promoting macrophages in the TME. PET imaging may thus provide a better impression of the physiology and anatomy of tumors and can guide physicians towards more tailored treatments for patients presenting TAM infiltration.

## Data Availability

Not applicable. No dataset was generated or analysed for this study and therefore, the data sharing is not applicable.

## Abbreviations

ABH: 2-(S)-amino-6-boronohexanoic acid
ApoE: Apolipoprotein E
Arg: Arginase
BBB: Blood–brain barrier
^11^C: Carbon-11
CCR2: C–C chemokine receptor type 2
CCL2: Chemokine (C–C Motif) ligand 2
CD: Cluster of differentiation
COX-2: Cyclooxygenase-2
CSF-1R: Colony stimulating factor 1 receptor
^64^Cu: Copper-64
DHFR: Dihydrofolate reductase
^18^F: Fluorine-18
FA: Folic acid
Fab: Antibody fragment
Fc: Fragment crystallizable region
FR: Folate receptor
^68^Ga: Gallium-68
^68^Ge: Germanium-68
Hp-Hb: Haptoglobin-hemoglobin
IC_50_: Half maximal inhibitory concentration
IFN-Ɣ: Interferon-gamma
IL: Interleukin
iNOS: Inducible nitric oxide synthase
KB: Human epithelial cancer cell
LPS: Lipopolysaccharide
MBq: Megabecquerel
MMR: Macrophage mannose receptor
Nb: Nanobody
OxLDL: Oxidized low-density protein
PET: Positron emission tomography
ROS: Reactive oxygen species
sdAb: Single-domain antibody
SPECT: Single-photon emission computerized tomography
SR: Scavenger receptor
SRCR: Cysteine-rich scavenger receptors
TAM: Tumor-associated macrophages
TGF-β: Transforming growth factor β
THF: Tetrahydrofolate
TME: Tumor microenvironment
TNF: Tumor necrosis factor
TNF-α: Tumoral necrose factor α
μGy: Microgray
μSv: Microsievert
